# Three-year evaluation of anthelmintic treatment efficacy against equine strongyles in France using FECRT

**DOI:** 10.1016/j.ijpddr.2026.100663

**Published:** 2026-07-24

**Authors:** Gabin Brucelle, Marie Delerue, Kenza Bourrier, Camille Gaudens, Sophie Lefebvre, Aurélie Merlin

**Affiliations:** a Anses, Laboratory for Animal Health in Normandy, Physiology and Epidemiology of Equine Diseases Unit, Goustranville, 14430, France; b Mixed Technological Unit “Equine Health and Welfare – Organisation and Traceability of the Equine Industry” (UMT SABOT), Goustranville, 14430, France; c French Horse and Riding Institute (IFCE), Development, Innovation and Research Department, Le Pin-au-Haras, 61310, France

**Keywords:** Equids, Strongyles, Anthelmintic resistance, Faecal egg count reduction test

## Abstract

Reduced anthelmintic efficacy against strongyles in equids is a widespread concern, but up-to-date field data are scarce. The aim of this study was to evaluate, over a three-year period, the efficacy of the anthelmintic (AH) molecules available in France (fenbendazole, pyrantel, ivermectin and moxidectin) against strongyles in equids using faecal egg count reduction tests (FECRTs). A total of 104 complete FECRTs (i.e., required sample size reached) were conducted in 98 groups, involving 749 equids kept in 70 facilities. Fenbendazole-resistant populations were detected in 8/9 groups (89%) from 7/8 facilities (88%; upper 90% CI range: 30.5%-83%) and pyrantel-resistant populations in 15/22 groups (68%) from 12/17 facilities (71%; upper 90% CI range: 56%-97%). Regarding macrocyclic lactone efficacy, ivermectin-resistant populations were identified in 14/51 groups (27%) kept in 11/41 facilities (27%; upper 90% CI range: 85.6%-99.9%) and moxidectin-resistant populations in 5/22 groups (23%) kept in 4/18 facilities (22%; upper 90% CI range: 85.3%-99.7%). Resistance was mainly detected in the Normandy region and in breeding farms (25/70), but also in riding schools offering boarding services (3/70), experimental platforms (2/70), and a zoological park (1/70).

In conclusion, this study provides the first evidence in France of resistance to all four available AH molecules and documents the spread of these resistances across the facilities. It also highlights the urgent need for regulatory changes requiring the use of AH only after a diagnostic assessment (e.g., targeted selective treatment based on faecal egg count results) in order to reduce the selective pressure exerted on parasite populations.

## Highlights

• Three-year evaluation of anthelmintic treatment efficacy against equine strongyles.
• Fenbendazole resistance was detected in 88% of facilities.
• Pyrantel resistance was detected in 71% of facilities.
• Ivermectin resistance was detected in 27% of facilities.
• Moxidectin resistance was detected in 22% of facilities.

## Graphical abstract

**Figure:**
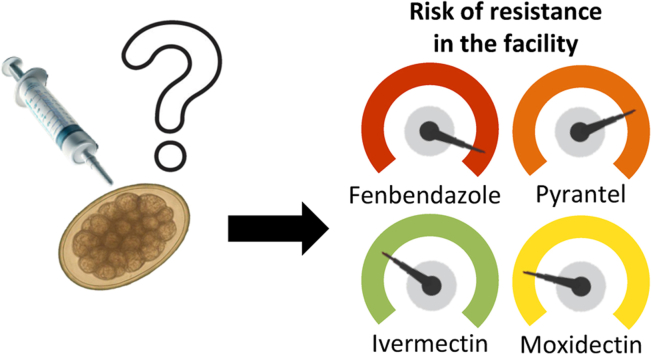

## 1 Introduction

Cyathostomins (Small Strongyles: Nematoda, Strongylida) are currently the most abundant gastrointestinal parasites of grazing equids regardless of age groups and the time of year (Collobert-Laugier et al., 2002; Corning, 2009). There are over 50 recognized species and co-infection with 15-20 different species is commonly encountered (Collobert-Laugier et al., 2002; Lichtenfels et al., 2008). Usually, cyathostomins are considered to be of low pathogenicity and most infected animals never show any clinical signs. However, the emergence of encysted larvae into the gut lumen, caused by various factors including seasonal influences, may induce larval cyathostominosis, resulting in sudden diarrhea, severe dehydration and serious colic, with mortality rates reaching up to 50% in young animals (Love et al., 1999; Corning, 2009).

To avoid any negative impact on equine health, French keepers rely on suppressive anthelmintic (AH) treatments administered several times a year, without specific risk assessment, using the following three licensed classes: i) benzimidazoles (fenbendazole-FENB), ii) tetrahydropyrimidine (pyrantel-PYR) and iii) macrocyclic lactones (ivermectin-IVM and moxidectin-MOX). Among these molecules, IVM is the most used drug by equine keepers of European countries (e.g., Osterman Lind et al., 2007b; Fritzen et al., 2010; Papini et al., 2015; Raś-Noryńska and Sokół, 2017; Dauparaitė et al., 2021; Merlin and Delerue, 2025). The frequency of deworming varied according to different factors such as the profile of the keepers (professionals of the equine sector > non-professionals), the age of the animals (foals > older animals) and breed groups (Thoroughbreds > other groups). Overall, keepers generally treat their foals every 1–3 months and their older animals every 3–6 months. Moreover, to assess each dose of AH, the weight of animal is rarely measured using a scale or a tape measure (Merlin and Delerue, 2025).

Unfortunately, frequent repeated use of the same class of AH and under-dosing promote the selection of resistant parasite populations (Sallé et al., 2017). The first cases of resistance in strongylids to FENB were reported in 1979, and resistance to pyrantel in 1991 (Barger and Lisle, 1979; Boersema et al., 1991). Since then, they have been widely documented in many countries and can now be considered well established. With regard to macrocyclic lactones, resistance in strongylids to this class of AH has been reported with increasing frequency over the past 19 years for IVM, and during the last six years for MOX (Campbell et al., 2007; Nielsen, 2022).

In France, the majority of studies that have evaluated the effectiveness of AH against strongylids have been carried out 9-15 years ago. They identified a widespread resistance to FBZ and some evidence of resistance to PYR (Traversa et al., 2012; Geurden et al., 2013; Sallé et al., 2017). More precisely, in two studies conducted on more than 15 french farms, resistance to FBZ was detected in 92%-94% of them (Traversa et al., 2012: in 17/18 farms; Sallé et al., 2017: in 36/39 farms), while resistance to PYR was reported in 10%-23% of farms (Traversa et al., 2012: in 3/30 farms; Sallé et al., 2017: in 9/39 farms). Recently, the first population of strongylids resistant to IVM, as well as to FENB and PYR was identified at a stud farm in Normandy (Merlin et al., 2024).

To prevent the development of resistance, the scientific community recommends selective treatment of adult equids based on strongylid faecal egg count (FEC). However, most surveys conducted in Europe, including France, indicate that only a small proportion of keepers (1-9%) regularly collect faeces in order to select adult equids for treatment (Osterman Lind et al., 2007b; Papini et al., 2015; Ras-Norynska and Sokoł, 2017; Dauparaitė et al., 2021; Merlin and Delerue, 2025). In contrast, higher levels of FEC use have been reported in some countries, such as Germany, Denmark and Sweden, with rates of 21%, 51% and 88%, respectively (Hinney et al., 2011; Nielsen et al., 2014; Hedberg Alm et al., 2023b).

Given the possible impact of these parasites on equine health, it is essential to monitor the effectiveness of AH in equids to be able to adapt and justify the recommendations regarding the use of AH to keepers. The objective of this study was to evaluate the efficacy of available molecules of AH against strongyles in French equids over a three-year period.

## 2 Materials and methods

### 2.1 Recruitment of facilities for the faecal egg count reduction test

The efficacy of the routine AH treatments planned by veterinarians in farms was assessed using the standardized method available, the faecal egg count reduction test (FECRT), following the last guidelines of the World Association for the Advancement of Veterinary Parasitology (WAAVP; Kaplan et al., 2023).

Equine facilities across France were recruited from July 2022 to September 2023 and from March 2025 to December 2025 using various channels: i) at trade fairs for professionals in the equine sector (e.g., “Normandy Horse meet'up”); ii) during a telephone campaign based on the public register of French breeders (https://www.ifce.fr/ifce/sire-demarches/donnees-sire/annuaire-des-naisseurs/); and iii) through citizen-science calls on the social networking platforms of the French Horse and Riding Institute (IFCE). Inclusion criteria were i) keeping at least one group of equids consisting of a minimum five animals, ii) no deworming of the animals in the previous two months, iii) grazing the animals together for at least three months to ensure that they were infected by the same parasite community, and iv) no change in the group composition during the test period (grazing together and no movement of equids outside the farm).

### 2.2 Anthelmintic molecules tested

Each tested AH treatment was orally administered by either the veterinarian or the farm keeper: i) FBZ paste (Panacur®, Intervet, Angers Technopole, Beaucouzé, France) at the recommended dose of 7.5 mg/kg body weight; ii) PYR paste (Strongid®, Zoetis, Malakoff, France) at the recommended dose of 6.6 mg/kg body weight or at a double dose of 13.2 mg/kg body weight, iii) IVM paste (Eqvalan® or Eqvalan Duo® or Furexel®, Boehringer Ingelheim Animal Health, Lyon, France; Equimax® or Eraquell®, Virbac, Carros, France; Nexmectin®, Audevard, Clichy, France; Alverin®, Zoetis, Chatillon, France) at the recommended dose of 0.2 mg/kg body weight, and iv) MOX paste (Equest® or Equest Pramox®, Zoetis, Chatillon, France) at the recommended dose of 0.4 mg/kg body weight. Appropriate individual AH dose was calculated based on the weight of each animal, either measured using a calibrated scale or estimated with the IFCE simulation tool from tape measurements (https://simulation.ifce.fr/poids_vif).

### 2.3 Faecal samples and analysis

To carry out each FECRT in a group of equids, individual faecal samples were collected on the ground from fresh droppings, immediately after defecation, on the day of AH administration (before treatment) or the day before, and a second set of samples was taken 14 days post-treatment.

After being kept at +4°C for 24h, faecal samples were first shipped in 2022 by express courier with ice packs to a departmental analysis laboratory (Qualyse®, Tulle, France), then in 2023 and 2025 to the laboratory for Animal Health (Normandy site, Anses). Upon arrival at the laboratory, all samples were stored at +4C° and analysed within four days of collection to reduce the effect of egg degradation (Nielsen et al., 2010).

In 2022 and 2023, strongylid faecal egg counts (FECs) were carried out using the modified McMaster technique (Raynaud, 1970). Five grams of faeces were diluted in 70 mL of sodium chloride solution with a relative density of 1.2 (two chambers read, sensitivity of 15 eggs per gram of faeces-epg). In 2025, FECs were performed using a more sensitive modified McMaster technique (Coles et al., 1992). Six grams of faeces were floated in 30 mL of sodium chloride solution (relative density = 1.2). Two McMaster slides (four grids) were examined, yielding a sensitivity of 12.5 epg.

No pre-test FECs could be performed, as recommended by the WAAVP, due to logistical constraints; animal keepers contacted us when they intended to treat their animals.

### 2.4 Required sample size to obtain a conclusive result

The minimum sample size required to obtain ≥80% power for each statistical test assessing resistance or susceptibility within an FECRT-research protocol, was calculated via the web application available at https://www.fecrt.com using i) the real arithmetic mean pre-treatment FECs, ii) the counting sensitivity of the coproscopic method used (15 epg in 2022-2023 and 12.5 epg in 2025), iii) the expected arithmetic mean efficacy for the AH used (FBZ: 99%; PYR: 98%; IVM and MOX: 99.9%), and iv) the lower efficacy threshold (FBZ: 95%; PYR: 88%; IVM and MOX: 96%) (Denwood et al., 2023; Kaplan et al., 2023).

FECRTs were stopped if the minimum sizes needed to achieve a statistical power of at least 80% for the resistance test were not reached.

### 2.5 Interpretation of FECRTs

The individual/mean FEC data of pre- and post-treatment were analysed using the web application (https://www.fecrt.com). The 90% upper and lower confidence intervals (CIs) of the percentage faecal egg count reduction at two weeks post-treatment were calculated using the Bayesian-Frequentist Hybrid Inference method (paired study design; research protocol; WAAVP method; Denwood et al., 2023). The 90% CIs were then compared to the target efficacy of the AH molecule being tested and its lower efficacy target, in order to classify each population of strongyles into three categories: i) Susceptible: when the lower 90% CI was greater than or equal to the lower efficacy threshold and the upper 90% CI was greater than or equal to the target efficacy; ii) Resistant: when the upper 90% CI was lower than the expected efficacy; iii) Inconclusive: if neither of these criteria were met. In case of inconclusive results, a less demanding FECRT-clinical protocol, requiring lower “lower efficacy thresholds” (FBZ: 90%; PYR: 80%; IVM and MOX: 92%), and consequently fewer animals and lower egg counts, was performed (Kaplan et al., 2023). However, the results were interpreted with greater caution due to the wider CIs.

When the post-treatment FECs were all zero, the Beta Negative Binomial (BNB) method (version A) was used to assess the effectiveness of the AH because the WAAVP method was unable to generate CI (Denwood et al., 2023).

### 2.6 Statistical analysis of the factors associated with the resistance

The statistical analyses were conducted with R version 4.5.1 (R Core Team, 2025).

#### 2.6.1 Assessment of the relationship between facility resistance status and facility characteristics

A generalized linear model (glm, link = logit) was implemented to investigate possible associations between facility resistance status (presence/absence of a population of strongylids resistant to at least one AH molecule) and facility characteristics: i) location (in Normandy/outside Normandy), type of facility (breeding farm/other type), main group of breeds (Thoroughbred/other group of breeds) and group of age (only animals ≤2 years old/only animals >2 years old/mixed age group). Before performing the model, each variable was tested for collinearity through a multicollinearity analysis (*vif()* function from the R car package, version 3.1.3) to ensure a variance inflation factor <5. The best model selection was performed using a backward stepwise approach based on the Akaike Information Criterion (*step()* function). Odds ratios (ORs) were calculated by exponentiating the beta coefficients from the final model, and 95% CIs were computed using the uniroots method (*Tidy()* function from the R broon package, version 1.0.11). Results were considered statistically significant when CIs did not include 1 and p < 0.05.

#### 2.6.2 Analysis of factors associated with the individual faecal egg count reduction after treatment

A generalized linear mixed model with a negative binomial distribution was fitted, through the glmmTMB package (version 1.1.14) to investigate the association between post-treatment individual FEC and the following fixed effects: i) sex (female/male), ii) age category (animals ≤2 years old/animals >2 years old), iii) breed (Thoroughbred/other breed), iv) location (in Normandy/outside Normandy), and v) type of facility (breeding farm/other type). The negative Binomial distribution was chosen to account for overdispersion and an excess of zero counts. Animals with zero pre-treatment FEC were excluded from the analyses.

In the model, farm was included as random effect, and pre-treatment individual FECs were log-transformed and included as an offset to account for baseline differences in egg excretion and to model post-treatment FECs relative to pre-treatment egg counts. Prior to model fitting, collinearity among variables was assessed. Model selection was performed using the backward stepwise method. Odds ratios were obtained by exponentiating the beta coefficients from the final model, and 95% CIs were calculated using the uniroots method (glmmTMB package). Results were considered statistically significant when CIs did not include 1 and p < 0.05. Nakawaga's R^2^ method was used to assess the variance explained by fixed and random effects in the final model (*r2_nakawaga()* function from the R performance package, version 0.17.0). Model validity was evaluated by verifying the absence of singular fits (*check_singularity()* function in R performance package), and by confirming model convergence (positive-definite Hessian matrix, glmmTMB package).

## 3 Results

### 3.1 Recruited facilities

A total of 95 facilities of equids were recruited. They were located in 85% of the 13 French metropolitan regions (11/13). The three main regions were Normandy (44% of facilities), Nouvelle-Aquitaine (12%) and Pays-de-la-Loire (9%). Concerning the type of facilities, 65% of them were breeding farms (62/95, including one offering also a horse boarding service), 22% riding schools (21/95, including 11 facilities with a horse boarding service), 4% exclusively horse boarding facilities (4/95), 4% experimental platforms (4/95), 2% educational establishments (2/95; agricultural college or rural family education center) and 2% zoological parks (2/95). Within all of these facilities, 140 groups of equids were included in the study (n = 1046 animals; mean of 1.5 groups per facility, min-max: 1-6).

### 3.2 Required sample size for a resistance test

Within the 140 groups, 149 FECRTs were carried out during the study period. However, 45 FECRTs were discontinued after the pre-treatment FECs (i.e., 44 groups kept in 37 facilities) because the required number of equids (determined by their egg excretion level, the sensitivity of the coproscopy technique used, and the AH molecule tested) to ensure a statistical power ≥80% for the resistance test (research protocol) was not reached (**Suppl. 1**). Consequently, the efficacy of an AH treatment could not be evaluated in 25 of the 95 recruited facilities (26%).

A total of 104 complete FECRTs (i.e., required sample size reached) were conducted in 98 groups, including 749 equids kept in 70 facilities. Among these tests, nine evaluated the efficacy of FBZ (2022: 1 FECRT, 2023: 6 FECRTs, 2025: 2 FECRTs), 22 evaluated the efficacy of PYR (2022: 3 FECRTs, 2023: 8 FECRTs, 2025: 11 FECRTs), 51 evaluated the efficacy of IVM (2022: 13 FECRTs, 2023: 24 FECRTs, 2025: 14 FECRTs), and 22 evaluated the efficacy of MOX (2022: 6 FECRTs, 2023: 4 FECRTs, 2025: 12 FECRTs).

### 3.3 Presentation of groups with complete FECRTs

Details of the 98 groups (70 facilities, 749 equids) with complete FECRTs are presented in **Suppl. 2**. Among these groups, 18% consisted of foals (18/98), 28% of yearlings (27/98), and 54% of animals older than two years old (53/98). In terms of species and breed composition, 39% of groups (38/98; 284 equids) were composed of mixed species and/or breeds (i.e., Saddle horses with ponies and/or racehorses and/or draft horses and/or donkeys), 22% of Thoroughbreds (22/98; 150 equids), 17% of saddle horses (17/98; 144 equids), 9% of ponies (9/98; 75 equids), 6% of draft horses (6/98; 52 equids), 4% of French trotters (4/98; 30 equids), 1% of donkeys (1/98; 8 equids) and 1% of zebras (1/98; 6 equids).

To evaluate AH treatment doses, animal weight was measured using a scale in 21 groups from eight facilities (i.e., 25 FECRTs), and estimated using the IFCE simulation tool based on tape measurements in 77 groups from 62 facilities (i.e., 79 FECRTs).

During the FECRTs, no animal showed clinical signs of parasitic gastroenteritis.

### 3.4 Summary of the effectiveness of the tested AH

All FECRT outputs are provided in **Suppl.3** (e.g., total number of eggs counted before treatment; required sample size, number of animals shedding eggs before and after treatment; confidence intervals, etc.).

Overall, one AH molecule was tested in 58 facilities, two molecules in 10 facilities and three molecules in two facilities. A strongyle population resistant to one AH molecule (FBZ, PYR, IVM or MOX) was identified in 50% of the facilities where a single AH was evaluated (29/58). Resistance to two AH molecules (FBZ + IVM) was found in one of the 10 facilities where two AH molecules were tested (a Thoroughbred stud farm), and resistance to three molecules (FBZ + PYR + IVM) in one of the two facilities where three molecules were tested (a Thoroughbred stud farm). Conversely, a strongyle population susceptible to three AH molecules (PYR, IVM, MOX) was found in one facility (a draught horse breeding farm).

**Fig. 1:**
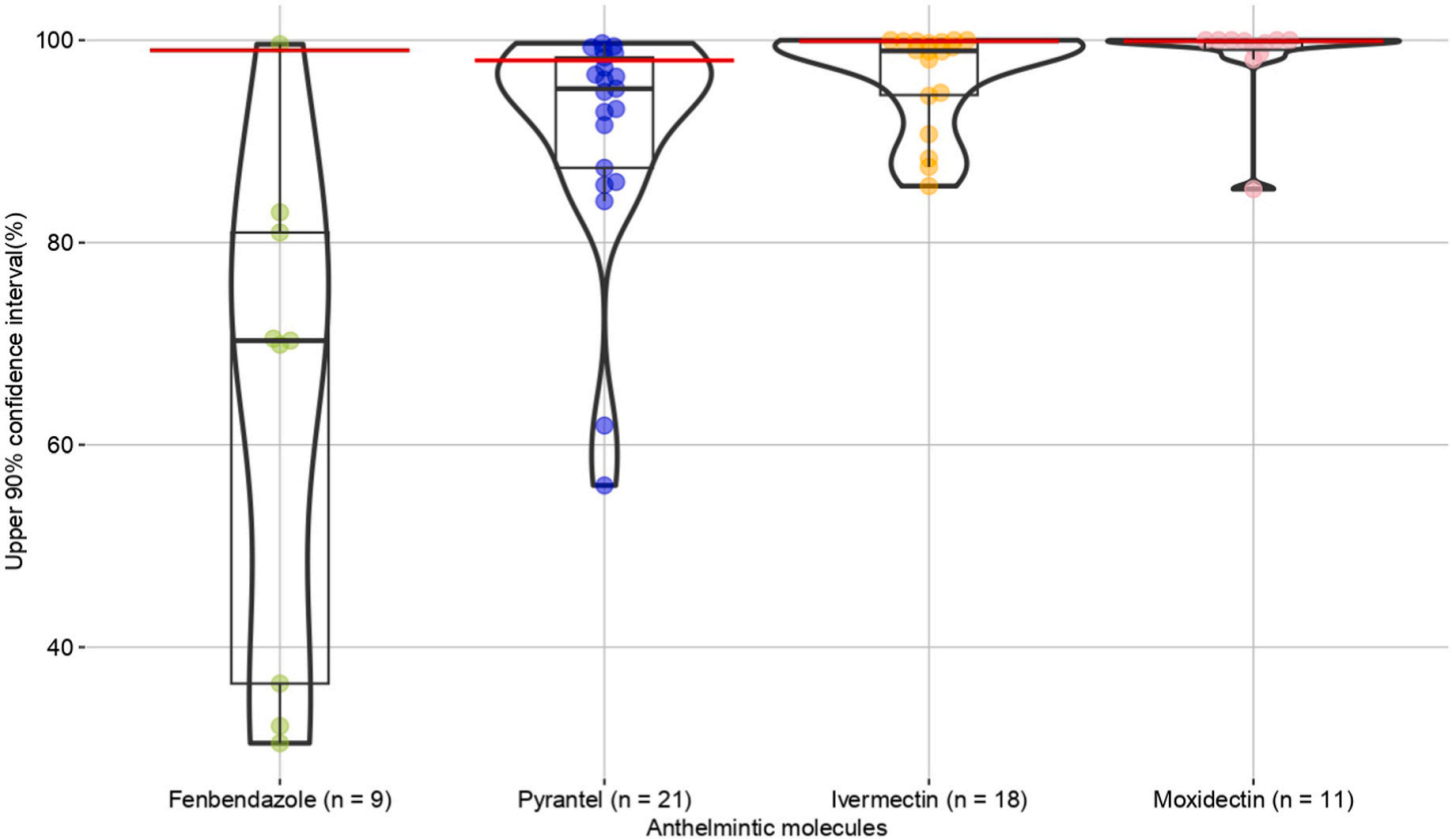
Distribution of the upper confidence interval calculated using the WAAVP method for each anthelmintic treatment between 2022 and 2025, compared with expected efficacy.

Regarding the efficacy of each AH molecule, a strongyle population resistant to FBZ was detected in 8/9 groups (89%) kept in 7/8 facilities (88%; upper 90% CI range: 30.5%-83.0%). In the remaining group, the result was inconclusive with the research protocol but susceptible with the clinical protocol (IC90%: [94.0%-99.6%]) (Fig. 1). Among the seven facilities where FBZ resistance was detected, three were Thoroughbred breeding farms (43%), two were riding schools offering boarding services (29%), one was a donkey breeding farm (14%), and one was a mixed-breed breeding farm (including ponies and saddle horses; 14%).

A strongyle population resistant to PYR was identified in 15/22 groups kept (68%) in 12/17 facilities (71%; upper 90% CI range: 56.1%-97.3%). Of these 15 groups, four (from three facilities) had received a double dose of pyrantel. A susceptible population to PYR was found in 6/22 groups (27%) in 4/17 facilities (24%); and for five of these groups, a 90% CI could be calculated using the WAAVP method (upper 90% CI range: 98.3%-99.7%). In the remaining group, no eggs were detected in post-treatment faeces (BNB method). Besides, one group (5%) yielded an inconclusive result with the research protocol but susceptible with the clinical protocol (90% CI: [87.0%-98.7%]). Among the 12 facilities where PYR resistance was detected, five (42%) were Thoroughbred breeding farms, two (17%) were experimental platforms, two (17%) were pony breeding farms, two (17%) were saddle horse breeding farms, and one (8%) was a French trotter breeding farm.

IVM-resistant populations were identified in 14/51 groups (27%) from 11/41 facilities (27%; upper 90% CI range: 85.6%-99.9%) and susceptible populations in 36/51 groups (71%) from 29/41 facilities (71%). Among these 36 susceptible groups, a 90% CI could be calculated for three using the WAAVP method (upper 90% CI range: 99.9%-100%). In the remaining 33 groups, no eggs were detected in post-treatment faeces (BNB method). Besides, one group (2%) yielded an inconclusive result with the research protocol but susceptible with the clinical protocol (2%; 90% CI: [96.8%-100%]).

Among the 11 facilities in which IVM resistance was detected, nine were breeding farms (82%), including five Thoroughbred farms, two saddle horse farms, one draught horse farm, and one pony farm. The remaining facilities were a riding school offering boarding services (9%) and a zoological park (9%).

Finally, MOX-resistant populations were identified in 5/22 groups (23%) from 4/18 facilities (22%; upper 90% CI range: 85.3%-99.7%), and susceptible populations in 16/22 groups (73%) from 13/18 facilities (72%). Among these 16 susceptible groups, a 90% CI could be calculated for five using the WAAVP method (90% CIs: 100%). In the remaining 11 groups, no eggs were detected in post-treatment faeces (BNB method). One group yielded inconclusive result with the research protocol but susceptible with the clinical protocol, with a 90% CI of 95.9%-99.9%. The four facilities where MOX resistance was detected were all breeding farms, comprising two Thoroughbred farms and two saddle horse farms (one of which also offered boarding services).

**Fig. 2:**
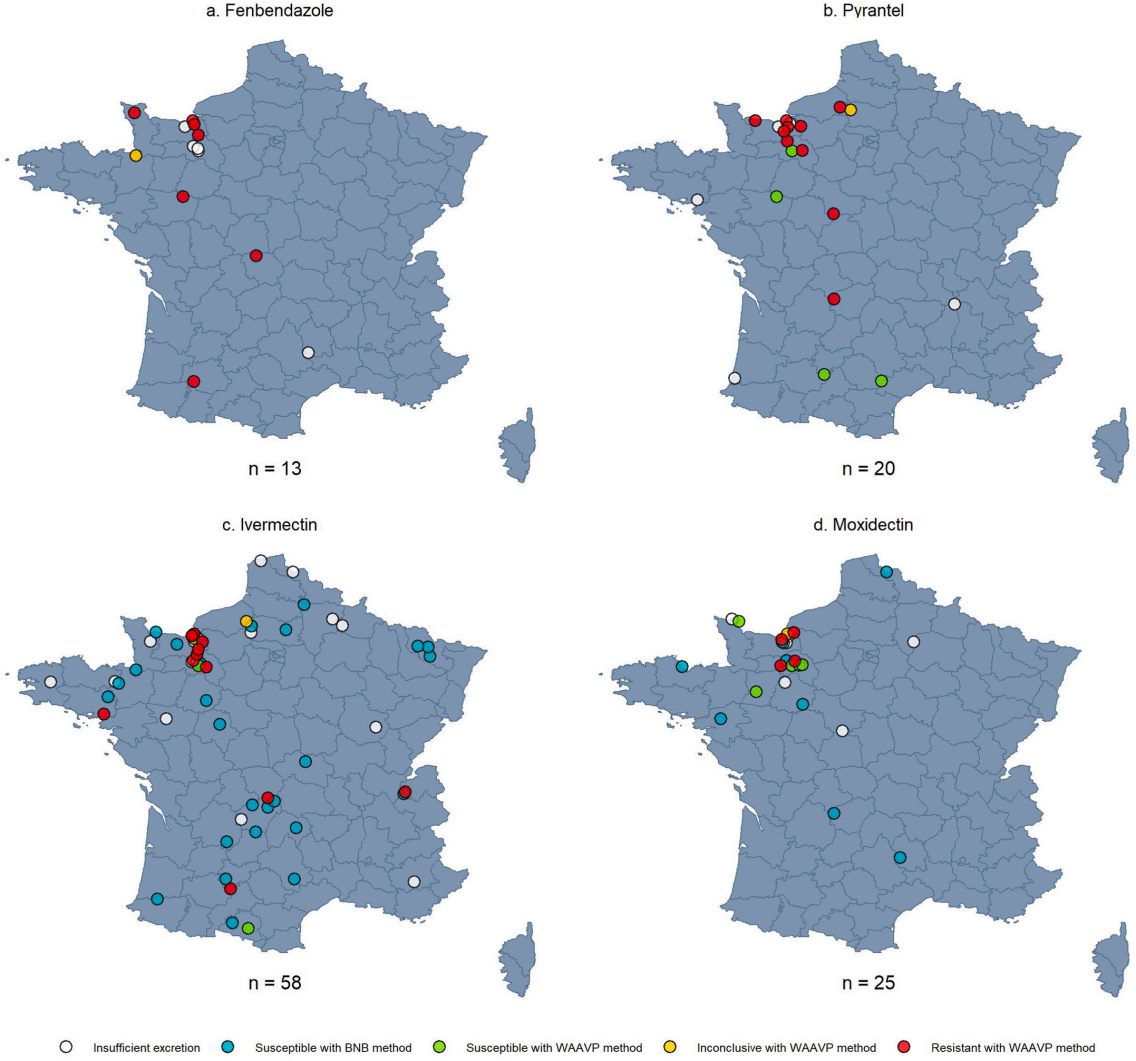
Geographical repartition of each faecal egg count reduction test (FECRT-research protocol) performed between 2022 and 2025. White dot, Insufficient excretion; blue dot, Susceptible with BNB method; green dot, Susceptible with WAAVP method; yellow dot, inconclusive with WAAVP method; red dot, resistant with WAAVP method.

The geographic distribution of the facilities with strongyle populations resistant to each AH molecule is presented in Fig. 2.

### 3.5 Monitoring the effectiveness of the same molecule over several years

The effectiveness of the same AH molecule was assessed in two different years (2022 or 2023 and 2025) in five facilities (12 groups). All facilities maintained the same status over time, remaining either susceptible (to IVM in one farm, MOX in one farm and PYR in one farm) or resistant (to MOX in one farm and PYR in one farm). In the facility with a PYR-resistant population, a single dose was administered to two groups of yearlings in 2023 (90% CIs: [−10.5%-91.6%] and [−7.6-96.6%]), whereas a double dose was given to two other groups of yearlings in 2025 (90% CIs: [−206.7%-61.9%] and [36.7-85.7%]).

### 3.6 Factors associated with the presence of at least one resistance in the facility

The variables “location” (p < 0.001) and “group of breeds” (p < 0.01) were significantly associated with the presence of a strongyle population resistant to at least one AH molecule in the facility. Resistance was less frequent in the facilities located outside Normandy than in those within this region (OR = 0.30, 95% CI = 0.09-0.96, p < 0.05). Furthermore, resistance was less frequent in facilities housing groups that included breeds other than Thoroughbreds compared with facilities keeping only Thoroughbreds (stud farms; OR = 0.11, 95% CI = 0.02-0.53, p < 0.05).

### 3.7 Factors associated with individual post-treatment faecal egg count

Individual FECs in equids before and after treatment are presented in **Suppl. 4**.

Among the animals, 32 animals with zero pre-treatment FEC were excluded from the generalized linear mixed model. Only the fixed effect “location” was significantly associated with the post-treatment FEC (p < 0.01). Animals located outside Normandy exhibited post-treatment FECs approximately 88 times lower than those in Normandy (OR = 0.011, 95%CI = 0.001-0.204, p < 0.01). In addition, 66% of the variance was explained by the random effect of the farm (Nakagawa's R2, conditional R2 = 0.94, marginal R2 = 0.28).

## 4 Discussion

The objective of this three-year study was to update knowledge on the level of efficacy of AH treatment against strongyles within a large French equine population. Strongyle populations resistant to all three licensed classes of AH were identified, specifically to the four molecules available: FBZ, PYR, IVM and MOX.

A higher risk of AH resistance was identified in facilities located in Normandy, France's leading horse-breeding region, with approximately 52,000 births each year (24% of national foal production; IFCE, 2025). This finding was consistent with the modeling results showing that resistance developed more quickly in yearlings than in adult equids (Leathwick et al., 2019). The lack of immunity in young animals will lead to a more rapid post-treatment increase in adult worm burden and faecal egg excretion, and thus to the presence of a greater number of worms carrying resistant genotypes that will be selected during the next AH treatment.

This study confirmed that the prevalence of strongylid resistance to FBZ in France remains high and comparable to values reported in previous surveys using different FECR methods (in 88% versus 92-94% of farms; Traversa et al., 2012; Sallé et al., 2017). The detection of FBZ-resistance has also been widely documented across Europe, with resistance detected in more than 70% of examined farms. Reported prevalence included 92% in Belgium (Dorny et al., 2000), 79% in Denmark (Craven et al., 1998), 85% in Germany (Traversa et al., 2009), 82% in Norway (Ihler, 1995), 75% in Romania (Cernea et al., 2015), 74% in Slovakia (Várady et al., 2000) and 72% in Sweden (Osterman Lind et al., 2007a). In contrast, lower prevalence levels have been reported in Italy (in 38% of farms; Traversa et al., 2009) and Ukraine (in 9% of farms; Kuzmina and Kharchenko, 2008), likely reflecting different patterns of FBZ use.

In our study, FBZ-resistances have been identified in breeding farms of various breed groups, as well as in riding schools offering boarding services. Riding schools offering boarding services can bring together equids owned by different individuals, each applying their own deworming protocol. As a result, animals within the same group may be treated at different frequencies, at different times, and with different AH molecules. Such heterogeneity can exert stronger selection pressure on parasite populations than that present in facilities where a single and coordinated protocol is applied to all equids within the group.

Despite this widespread resistance, FBZ is still used by 57% of French keepers for equids older than one year (Merlin et al., 2025). It would therefore be appropriate to re-examine the relevance of using this molecule for the treatment of strongylid infections.

Regarding PYR, this molecule is the least commonly used in France (administered by 39% of keepers in adult equids; Merlin and Delerue, 2025). However, resistance to PYR now appears to be more frequent across facilities, being detected in 71% of them, compared with 10-23% of farms in earlier French studies (Traversa et al., 2012; Sallé et al., 2017).

Since the early 1990s in the Netherlands (Boersema et al., 1991), reports of PYR resistance have been received from many European countries, but most often with a prevalence ≤30% for studies including more than 15 farms (e.g., Denmark: 12 farms with resistance out of 64, Nielsen et al., 2013; Germany: 4 out of 20, Traversa et al., 2009; Italy: 18 out of 60, Traversa et al., 2009; Lithuania: 5 out of 25, Dauparaitė et al., 2021) with the exception of a recent study conducted in Sweden (9 farms with resistance out of 16, Hedberg Alm et al., 2023a). Given that most European studies are now relatively dated, it would be relevant to undertake new investigations to assess the current evolution of PYR efficacy.

In our study, populations of strongylids resistant to PYR were detected not only in farms raising different breeds, but also in experimental platforms that have been rationalizing the use of AH treatments for several years (e.g., administering PYR once a year in summer and only to animals shedding more than 500 eggs of strongyles per gram of faeces).

Ivermectin is the molecule used every year by almost all equine keepers, and sometimes even several times a year in young animals (Merlin and Delerue, 2025). It was therefore unsurprising that keepers more frequently requested an assessment of IVM efficacy.

The first strongyle population resistant to IVM was identified in a Thoroughbred stud farm in Normandy, which is included in this study (Merlin et al., 2024). Following this initial detection, additional resistant populations were found in four other Thoroughbred farms (i.e., a total of 5 stud farms with resistance out of the 9 recruited), as well as in other types of breeding farms (2/8 saddle horse farms, 1/3 draught horse farms and 1/2 pony farms). Resistance was also detected in a riding school (1/11, i.e. 9%) and in a zoological park (1/1, i.e. 100%). In zoological parks, handling wild animals can be challenging. It would therefore be advisable to reconfirm this result to ensure that no errors in weight estimation (leading to underdosing) or issues related to drug administration occurred.

Resistant populations to IVM have already been reported in a few other European countries, notably in Finland in trotter farms (Näreaho et al., 2011), in Italy (farm type not specified, Traversa et al., 2009), and in the United Kingdom in Thoroughbred farms (Bull et al., 2023). In Denmark and Sweden, where faecal examination-based selective treatment has been supported by legislation since 1999 and 2007, respectively, no signs of IVM resistance have been reported (Larsen et al., 2011; Hedberg Alm et al., 2020, 2023a).

The first strongyle populations resistant to MOX were identified in Normandy in 2 out of the 3 Thoroughbred stud farms recruited and in 2 out of 4 saddle horse breeding farms sampled. Resistances to MOX have also been identified in Thoroughbred stud farms in the United States (following the importation of yearlings from Ireland; Nielsen et al., 2020), Australia (Abbas et al., 2021) and more recently in England (Bull et al., 2023). It would be interesting to test the efficacy of IVM in facilities with MOX-resistant strongyle populations to assess whether the mutations responsible for MOX resistance also confer resistance to IVM. Indeed, the MOX-resistant strongyle populations identified in the United States and England were also resistant to IVM (Nielsen et al., 2020; Bull et al., 2023).

Longitudinal monitoring of AH treatment efficacy conducted in five facilities showed that the sensitivity or resistance status of the strongyle population remained stable over a period of two to three years. This observation raises the question of whether it is useful or relevant to carry out a FECRT every year in a facility where the parasite population is still sensitive. Leathwick et al. (2019), using a mathematical modelling approach, demonstrated that in facilities located in temperate regions where all yearlings are dewormed 2 to 4 times per year, the first IVM-resistant strongyle populations may emerge within only five to ten years. Given this relatively rapid evolutionary timescale, and considering that the duration for which each owner has been applying their deworming protocol is generally unknown, irregular monitoring increases the risk of failing to detect the early stages of resistance development. Implementing an annual FECRT for each molecule would therefore constitute a more prudent and relevant approach, as it would allow rapid adjustments to deworming protocols and help limit the dissemination of resistant genotypes during the movement of equids between facilities.

Once resistance is established within a facility, it tends to persist and may even strengthen over time. This pattern was observed in a Thoroughbred stud farm, where the upper 90% CI continued to decline following PYR treatments over a two-year period, despite the administration of a double dose during the second FECRT in 2025. These observations suggest that, once strongyles have developed resistance to PYR, increasing the dose not restore therapeutic efficacy. Implementing annual FECRT, even in facilities where resistant populations are already present, is therefore essential for monitoring the progressive loss of efficacy of the molecules concerned.

The generalized linear mixed model revealed a strong farm effect on individual post-treatment FEC. It would therefore be valuable to conduct surveys within these facilities to characterize management practices and to confirm factors previously identified as being associated with an increased risk of resistance (e.g., frequent deworming, underdosing, absence of quarantine protocols when introducing new animals; Sallé et al., 2017), as well as to identify potential other factors. However, due to technical constraints, notably limited personnel resources and the large number of farms included, it was not possible to collect such information in the present study.

This study was based on the WAAVP's FECRT protocol, which can be difficult to apply for several reasons such as it cannot be used in small groups (<5 animals), and it requires a minimum level of egg excretion even when groups include more than five shedding equids. As a result, it was not possible to perform a FECRT-research protocol in 26% of our recruited facilities (25/95). However, additional slides could have been examined from each animal in the pre-treatment groups, or repeated FECs could have been performed until reaching egg counts exceeded the required threshold. A more sensitive FEC method, such as the Mini-FLOTAC technique (sensitivity: 5 epg), could also have been used. Besides, within a context of veterinary monitoring, it would have been possible to perform a FECRT-clinical protocol in 9/25 facilities (36%).

To enable broader evaluation and surveillance of anthelmintic efficacy, it is therefore essential to develop alternative diagnostic approaches, such as molecular methods for detecting resistance, and to establish a consensus guideline among parasitologists for a semi-quantitative assessment of deworming efficacy (e.g., by comparing the number of egg-shedding animals before and after treatment). Such a semi-quantitative FECRT would allow each group of equids to be classified according to its level of resistance risk (from low to high suspicion of a resistant strongylid population) and would facilitate comparisons across studies and countries. These alternative tools are especially important given that most French keepers have fewer than five equids (Farchati et al., 2021).

## 5 Conclusion

This study, conducted across a large number of facilities over a three-year period, enabled the identification for the first time of strongyle populations resistant to all available AH molecules, and documented the extent of their dissemination. It further highlights the urgent need to rationalize the use of AH treatments by implementing selective-treatment strategies that maintain a strongyle population in refugia and reduce selection pressure. In France, only 5% of equid keepers implement faecal egg count-based targeted selective treatment, despite the dissemination of guidelines and scientific articles aimed at veterinarians, as well as repeated messaging during professional and socio-professional conferences (Merlin and Delerue, 2025). The warnings and recommendations issued by the scientific community over the past decade have not been sufficient to modify the practices of veterinarians and keepers. Regulatory action now appears to be the only means capable of driving a durable shift in management practices.

## Funding sources

This work was supported by the French Agency for Food, Environmental and Occupational Health & Safety (Anses).

## CRediT authorship contribution statement

**Gabin Brucelle:** Data curation, Investigation, Methodology, Validation, Writing – original draft. **Marie Delerue:** Validation, Writing – original draft. **Kenza Bourrier:** Data curation, Investigation, Validation. **Camille Gaudens:** Data curation, Investigation, Validation. **Sophie Lefebvre:** Data curation, Investigation, Validation. **Aurélie Merlin:** Conceptualization, Data curation, Formal analysis, Funding acquisition, Investigation, Methodology, Project administration, Supervision, Validation, Writing – original draft.

## Declaration of interest

The authors declare no conflicts of interest with respect to the research, authorship, publication of this article and/or financial and personal relationships that could inappropriately influence this work.
